# The causal mutation in *ARR3* gene for high myopia and progressive color vision defect

**DOI:** 10.1038/s41598-023-36141-0

**Published:** 2023-06-02

**Authors:** Lei Gu, Peikuan Cong, Qingyao Ning, Bo Jiang, Jianyong Wang, Hongguang Cui

**Affiliations:** 1 The First Affiliated Hospital, Zhejiang University School of Medicine, Hangzhou, China; 2 Key Laboratory of Growth Regulation and Translational Research of Zhejiang Province, School of Life Sciences, Westlake University, Hangzhou, China

**Keywords:** Clinical genetics, Clinical genetics

## Abstract

The *ARR3* gene, also known as cone arrestin, belongs to the arrestin family and is expressed in cone cells, inactivating phosphorylated-opsins and preventing cone signals. Variants of *ARR3* reportedly cause X-linked dominant female-limited early-onset (age < 7 years old) high myopia (< − 6D). Here, we reveal a new mutation (c.228T>A, p.Tyr76*) in *ARR3* gene that can cause early-onset high myopia (eoHM) limited to female carriers. Protan/deutan color vision defects were also found in family members, affecting both genders. Using ten years of clinical follow-up data, we identified gradually worsening cone dysfunction/color vision as a key feature among affected individuals. We present a hypothesis that higher visual contrast due to the mosaic of mutated *ARR3* expression in cones contributes to the development of myopia in female carriers.

## Introduction

Globally, myopia is the most common refractive error with a prevalence rate of approximately 30% but climbing rapidly, and its prevention is becoming an urgent problem^1,2^. Deep understanding of the mechanism of myopia development is key to effective prevention. Hereditary myopic diseases can provide the genes or genetic loci whose functions are associated with myopia development. Currently, more than 500 genes as well as 25 genetic loci associated with myopia have been identified^3^.

*ARR3* is an early-onset high myopia (eoHM) causal gene, located on the X-chromosome but known for its female-limited nature. Xiao et al. first reported in 2016 three cases of female-limited eoHM with heterozygous mutation of the *ARR3* gene^4^. Another Chinese pedigree carrying *ARR3* mutation with similar clinical presentation was reported in 2020^5^. Széll et al. reported a non-Chinese eoHM mutation family in Hungary with new *ARR3* mutation and with clinical features similar to those of affected females with eoHM and color vision defect^6^. So, *ARR3* is a relatively rare mutation loci that occurs worldwide and is strongly associated with female-limited eoHM clinical manifestation^7^.

*ARR3* belongs to the arrestin protein family. The arrestin family is a group of highly conserved proteins with four members in mammals: arrestin 1 (visual or rod arrestin, also called S-antigen, *SAG*), β-arrestin 1 (*ARRB1*, also called arrestin-2), β-arrestin 2 (*ARRB2*, also called arrestin-3) and arrestin 3 (cone arrestin, *ARR3*, also called arrestin-4)^8^. Arrestin proteins binding to G-protein-coupled receptors (GPCRs) block G protein interactions and redirect signaling to numerous G-protein-independent pathways^9^. The family can be subdivided into two subfamilies structurally and functionally: visual (cone and rod arrestin) and non-visual (β-arrestin1 and β-arrestin 2)^10^. *ARR3* is highly expressed in cone cells and pinealocytes, but details of its function in pinealocytes remain unknown^8–10^. In single living cones, *ARR3* protein binds to light-activated phosphorylated opsins, then desensitizes them and stops their continuous stimulations. *ARR3* knockout mouse models have shown no significant difference in cone signal shutoff compared to controls in native murine cones, but have shown a decrease in contrast sensitivity^11,12^.

Here, we report a long-term follow-up study of a multigeneration family with a new *ARR3* causal mutation, identifying eoHM phenotype in seven affected females, late-onset high myopia in one affected male, and protan/deutan color vision defect in seven individuals of both genders.

## Materials and methods

### Subjects

**Figure 1:**
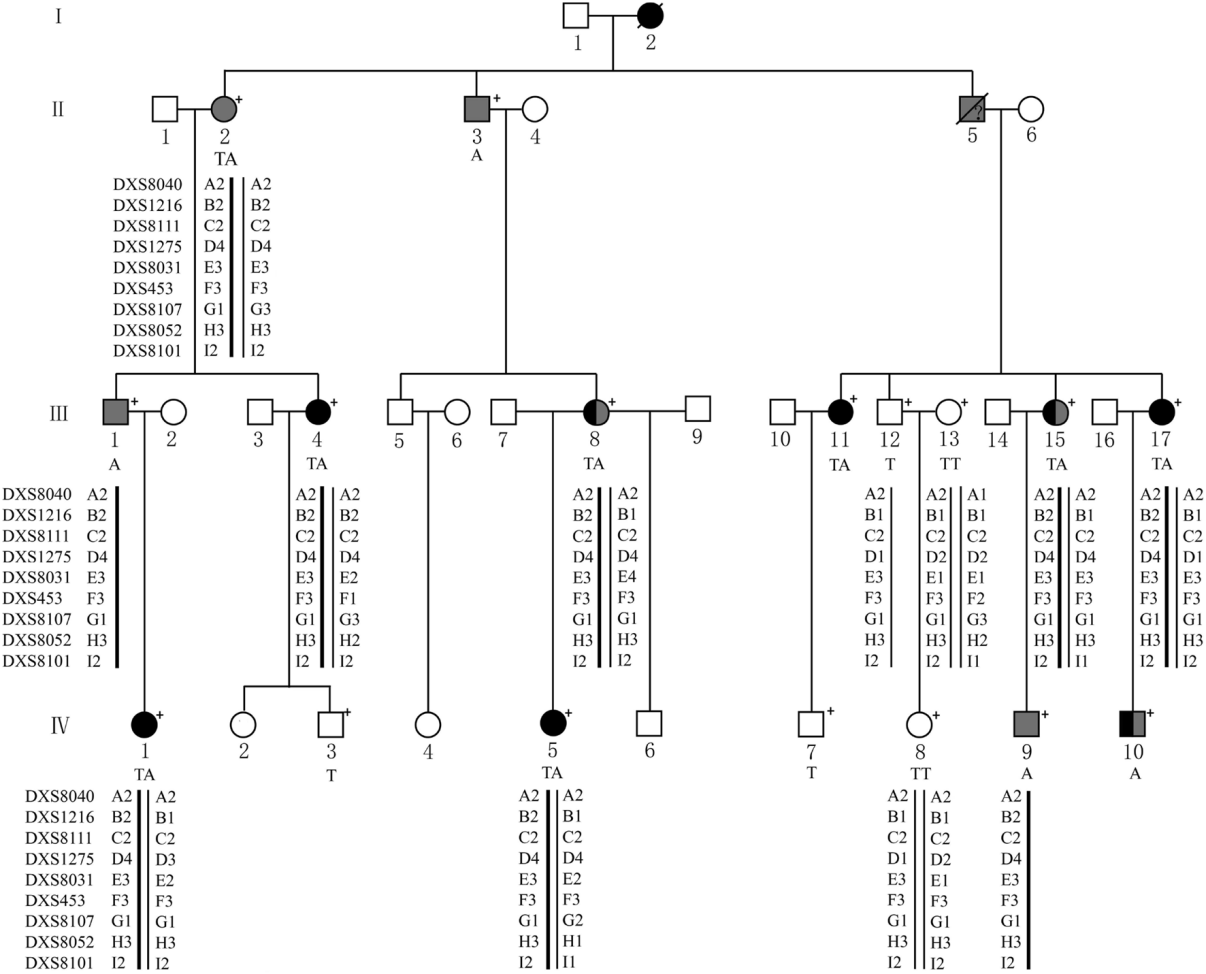
Pedigree of the family affected by X-linked dominant high myopia and defective color vision. Only IV-10 had late-onset high myopia, arising in his middle school years, other high myopia patients were all early-onset high myopia (eoHM). Gray color: protan/deutan color vision defect; black color: high myopia. ?: uncertain manifestation. +: available family members.

This study focused on four generations of a Chinese family (Fig. 1) from Eastern China. They were recruited at the Department of Ophthalmology, the First Affiliated Hospital Zhejiang University School of Medicine. Informed written consent was obtained from all subjects, following the Declaration of Helsinki. This study was approved by the Institutional Review Board of the First Affiliated Hospital Zhejiang University School of Medicine and all experiments were performed in accordance with relevant guidelines and regulations. All participants underwent a comprehensive standardized examination by ophthalmologists, including visual acuity, color vision (pseudoisochromatic plates and RGB anomaloscope), slit lamp, funduscopic examination, intraocular pressure, IOL-Master, optical coherence tomography (OCT) and confocal-scanning laser fundus autofluorescence (FAF, Heidelberg Engineering, Heidelberg, Germany), full-field flash electroretinography (ERG, Roland Consult GmbH, Brandenburg, Germany) and ocular imaging prior to genetic testing. ERG test includes the data from five channels: rod response (dark adapted 0.01 cd-s-m^−2^), maximal mixed retina response (dark adapted 3.0 cd-s-m^−2^), oscillatory potentials, cone response (light adapted 3.0 cd-s-m^−2^) and 30 Hz flicker (light adapted flicker 3.0 cd-s-m^−2^). Refractive error and the radius of corneal curvature in the horizontal and vertical meridian were measured using an autorefractor (KR8800, Topcon, Tokyo, Japan). The diagnosis for high myopia in this study required a spherical equivalent of − 6.0D or below for both eyes. Early-onset high myopia was defined as development of high myopia before the age of school years^13^. Detailed physical examination was performed and general medical and past ophthalmic histories were acquired to exclude any known syndromic forms of myopia, such as Marfan syndrome, Stickler syndrome, or Knobloch syndrome. Genomic DNA was extracted from peripheral blood following standard procedures.

### Whole exome sequencing

II-1, II-3, III-15, IV-1, IV-2 and IV-5 in the family were sequenced on Illumina HiSeq 4000 platform with whole exome sequencing by the Beijing Genomics Institute. The original reads were aligned to the human reference genome assembly (GRCh37). Burrows-Wheeler Aligner^14^ and Genome Analysis Toolkit^15^ were used to analyze the sequencing data following standard procedures. Variants were annotated using ANNOVAR^16^.

### Validation and screening of *ARR3* mutation

Genomic DNA from all the available members in the family and 150 unrelated female sporadic Chinese cases with high myopia were isolated from peripheral blood using RelaxGene Blood Kit (TIANGEN, Beijing, China), following the manufacturer’s instructions. The exons and exon–intron boundaries sequence of *ARR3* gene were downloaded from Ensembl Genome Browser. The primers were designed using the Primer-BLAST (Table S1). All the coding and splice site regions of *ARR3* gene were sequenced for all individuals. The data were analyzed by using Mutation Surveyor software (SoftGenetics, USA).

### Screening of *OPN1LW *and *OPN1MW* mutation

The basic structure of *OPN1LW* and *OPN1MW* gene cluster was captured and amplified by PCR, then screened with Sanger sequencing as previously described^17,18^. Briefly, *OPN1LW* and *OPN1MW* ’s exons were captured using specifically designed primers as first step (Tables S2 and S3), then amplified for Sanger sequencing using Step 2 primers shown in Table S3. The data were analyzed by using Mutation Surveyor software (SoftGenetics, USA).

### Linkage analysis

Confirmatory linkage analysis was performed using DNA from 13 family members. Nine microsatellite markers flanking the *ARR3* gene were selected to study co-segregation in the family, including *DXS8040*, *DXS1216*, *DXS8111*, *DXS1275*, *DXS8031*, *DXS453*, *DXS8107*, *DXS8052* and *DXS8101*. The primers for each microsatellite marker were downloaded from UniSTS (NCBI). The amplified polymerase chain reaction (PCR) products were analyzed on 8% non-denaturing polyacrylamide gel and visualized by silver staining.

## Results

### Subjects

**Figure 2:**
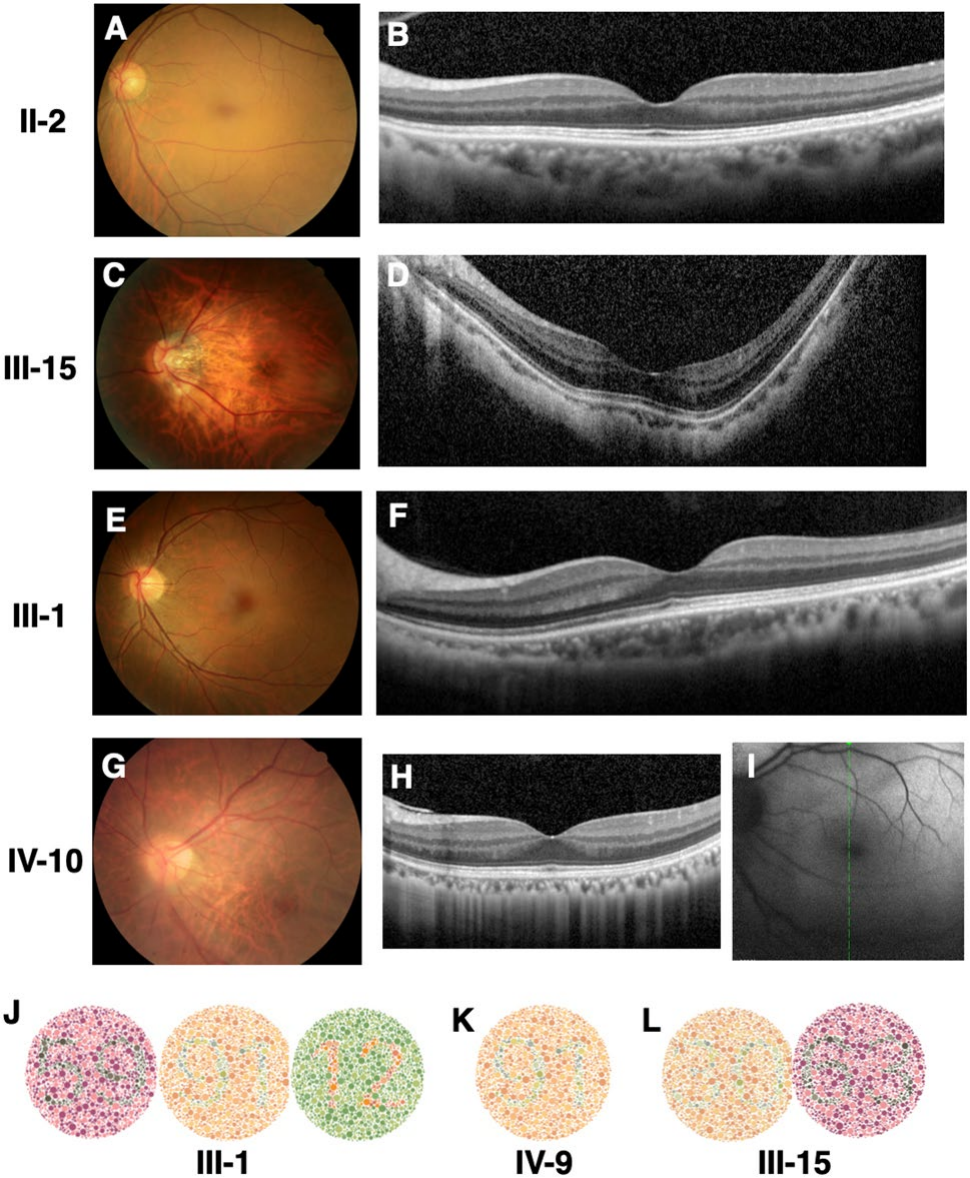
Fundus features of two female (II-2 and III-15) and two male carriers (III-1 and IV-10), who all had defective color vision. Color fundus photos (**A**,**C**,**E**,**G**), OCT images (**B**,**D**,**F**,**H**) and auto fluorescent fundus photo (**I**) for left eyes. III-15 and IV-10 had high myopia. The mis-recognized pseudo isochromatic plates of III-1 (**J**), IV-9 (**K**), and III-15 (**L**) in color vision tests.

The pedigree of the Han Chinese family with X-linked dominant high myopia and color vision defect is shown in Fig. 1. Contact with IV-6 was not possible due to parental divorce. Detailed clinical data in affected members are summarized in Table 1. Seven affected female patients within the family were diagnosed with eoHM myopia, with onset before school age and stable myopic level during ~ 10 years of follow-up. The spherical equivalent of affected females ranged from -8.0 to -24.3 diopters, and axial length ranged from 25.4 to 31.0 mm. The female carrier II-2 was emmetropic. Four patients with very high myopia (worse than -10.0D; III-8, III-11, III-15 and III-17) showed typical fundus features of high myopia: thinning of the retinal pigment epithelium (RPE) and the choriocapillaris, providing a ‘tigroid’ or ‘tessellated’ fundus appearance (Fig. 2). IV-10 was the only male carrier with high myopia, with onset age ~ 12 years, in contrast to the phenotype of female patients in the family, considering Mendelian myopia. The male carrier III-1 had low myopia with onset age > 12 years. The other male carriers (II-2 and IV-9) were emmetropic. OCT results in all affected members showed continuous but mildly thinned photoreceptor layers, and the individual with high myopia showed features associated with myopia, including significantly thinned retinal layers, and posterior scleral staphyloma (Fig. 2C,D). III-11 had macular retinoschisis in her left eye. Results of FAF were normal in all affected individuals, without the hyper-reflective sign characteristic of cone dystrophy.

**Table 1:** Clinical characters of all available affected individuals with ARR3 mutation.

Subject no	Gender	Age (years)	Eye	Axial length (mm)	Refraction (DS)	Best corrected visual acuity	Color vision	Fundus morphologic findings
II-2	F	56	OD	21.8	+ 1.6	NA	NA	Normal
			OS	22.0	+ 1.3	NA		Normal
		65	OD	21.78	+ 2.25	20/30	Color vision defect	Normal
			OS	22.0	+ 2.25	20/25		Normal
II-3	M	68	OD	24.17	NA	NA	Color vision blindness	Proliferative diabetic retinopathy, macular edema
			OS	23.64	NA	NA		Proliferative diabetic retinopathy, macular edema
III-1	M	35	OD	25.5	− 3.8	NA	Color vision defect	Normal
			OS	25.3	− 2.8	NA		Normal
		40	OD	25.6	− 3.75	20/30	Color vision defect	Normal
			OS	25.4	− 3.25	20/30		Normal
III-4	F	35	OD	25.6	− 10.1	NA	NA	Conus
			OS	26.2	− 9.8	NA		Conus
		44	OD	25.49	− 6.75	20/33	Normal	Conus, tigroid
			OS	26.33	− 8.00	20/30		Conus, tigroid
III-8	F	47	OD	28.77	− 13.25	20/100	Color vision defect	Tigroid, conus, Fuchs spot
			OS	28.59	− 13.25	20/60		Tigroid, conus, Fuchs spot
III-11	F	40	OD	30.5	− 24.3	NA	NA	Tigroid, conus
			OS	29.7	− 21.9	NA		Tigroid, conus
		47	OD	30.99	− 25.00	20/60	Normal	Tigroid, conus
			OS	30.00	− 23.75	20/100		Tigroid, conus, macular retinoschisis
III-15	F	46	OD	30.0	− 18.5	NA	NA	Tigroid, conus, Fuchs spot
			OS	29.0	− 16.4	NA		Tigroid, conus, Fuchs spot
		53	OD	30.37	− 19.00	20/130	Color vision defect	Tigroid, conus, Fuchs spot
			OS	29.23	− 14.00	20/100		Tigroid, conus, Fuchs spot
III-17	F	49	OD	29.5	− 21.8	NA	NA	Tigroid, conus, Fuchs spot
			OS	29.6	− 21.8	NA		Tigroid, conus, Fuchs spot
		55	OD	29.54	− 24.75	20/200	Normal	Tigroid, conus, Fuchs spot
			OS	29.65	− 23.25	20/160		Tigroid, conus, Fuchs spot
IV-1	F	8	OD	24.4	− 8.4	20/20	Normal	Normal
			OS	24.1	− 8.0	20/20		Normal
		14	OD	25.22	− 7.00	20/20	Normal	Normal
			OS	24.90	− 6.50	20/20		Normal
IV-5	F	11	OD	NA	− 5.0	NA	NA	NA
			OS	NA	− 6.0	NA		NA
		20	OD	NA	− 7.0	20/20	Normal	NA
			OS	NA	− 7.0	20/20		NA
IV-9	M	18	OD	23.6	+ 1.3	NA	NA	Normal
			OS	23.7	0	NA		Normal
		31	OD	24.1	− 0.75	20/22	Color vision defect	Normal
			OS	24.03	− 0.5	20/22		Normal
IV-10	M	34	OD	25.01	− 7.5	20/20	Color vision defect	Conus
			OS	25.63	− 11.5	20/22		Conus

### Color vision tests findings

Patient II-3 reported a significant decline in color vision over the past two years, and at the end of follow-up had failed to recognize any pseudoisochromatic chart, difficult to follow RGB anomaloscope test, indicating protan/deutan color blindness. He was diagnosed with diabetes mellitus more than ten years previously and at the end of follow-up had proliferative diabetic retinopathy and macular edema, so the cause of decline in his vision may be multifactorial. Individuals III-1 and IV-10 were first diagnosed with defective color vision in high school and could not read numbers in several pseudoisochromatic chart (Fig. 2J). IV-9 had normal color vision test results when first enrolled in this study at 22 years of age, and showed a mild protan/deutan color vision defect with one mis-recognized pseudoisochromatic chart “91” read as “01” at the final follow-up visit (Fig. 2K). Four female carriers (II-2, III-8, III-15 and III-17) failed pseudoisochromatic color vision testing (Fig. 2L). All family members performed well on the RGB anomaloscope test except II-3.

### Electroretinography findings

**Figure 3:**
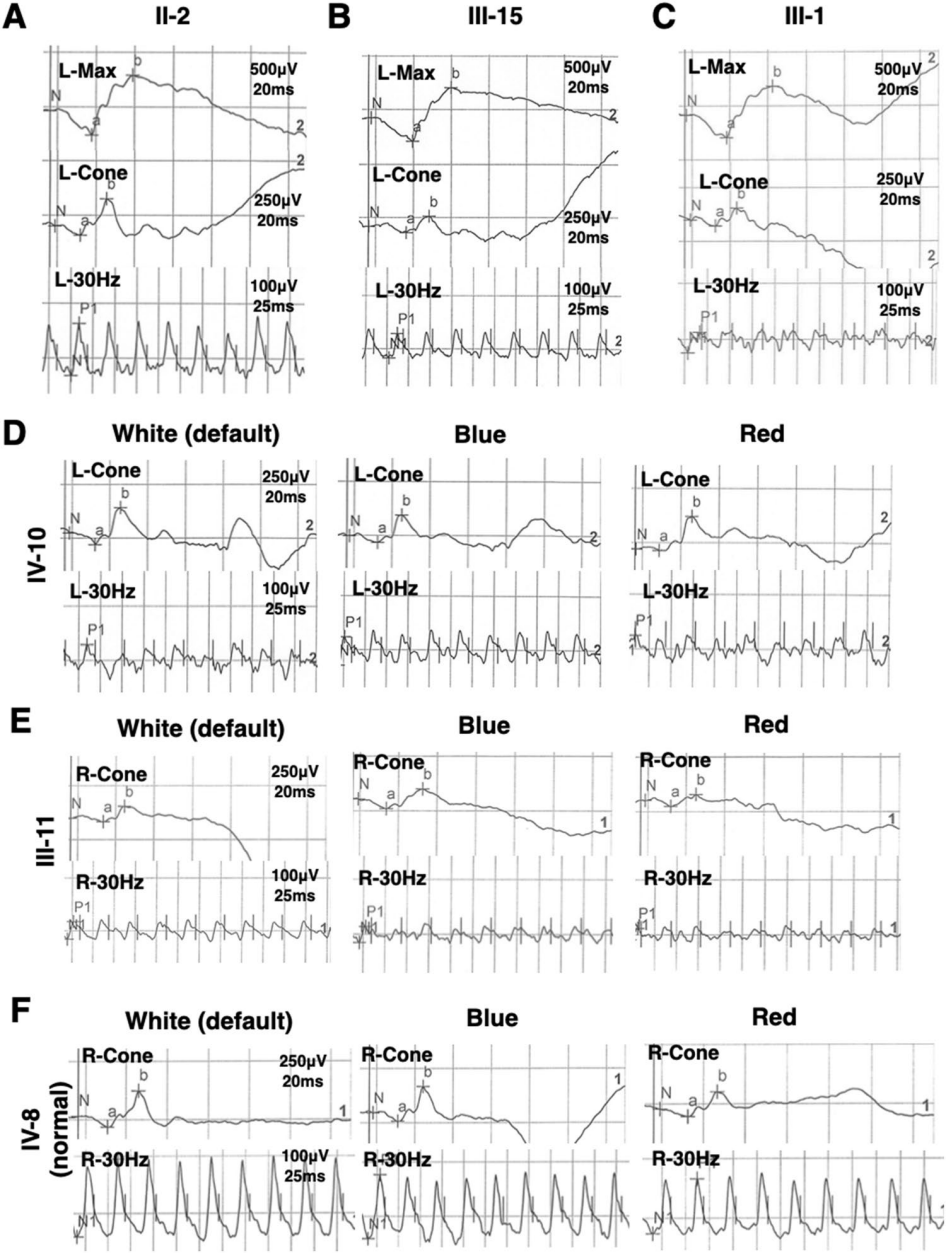
Electroretinography (ERG) recordings of family members. (**A**) II-2, 65 year-old female patient with color vision defect only. (**B**) III-15, 43 year-old female patient with early-onset high myopia and color vision defect. (**C**) III-1, 32 year-old male patient with color vision defect only. (**D**) IV-10, 32 year-old male patient with Mendelian high myopia and color vision defect. (**E**) III-11, 46 year-old female with early-onset high myopia only. (**F**) IV-8, 16 year-old female normal family member as normal control. L-, left eye. R-, right eye. Max, dark-adapted 3.0 maximal combined rod-cone response. Cone, light-adapted 3.0 single-flash photopic ERG of cone response. 30 Hz, light-adapted 3.0 flicker ERG. White, default flash full-field ERG. Blue, blue flash full-field ERG. Red, red flash full-field ERG.

White full-field electroretinography (ERG) was normal in the 65-year-old female carrier II-2 with color vision defect only (Fig. 3A). III-15 (43 year-old female with both high myopia and color vision defect) had mildly reduced amplitudes in all channels with responses typical of high myopia (Fig. 3B)^19^. III-1 (32 year-old male), with only color vision defect, showed abnormal responses to 30-Hz flicker stimuli with lower amplitude, indicating abnormal function of cone cells (Fig. 3C).

Red color flash ERG is a specialized procedure used in addition to standard white flash ERG to distinguish the function of cone cells, especially L- and M-cones, helping to determine the origins of abnormal standard ERG^20,21^. Blue color flash ERG is sensitive to the function of rod and S-cone cells. IV-10 (32 year-old male, with both late onset high myopia and color vision defect) had normal rod response but mildly abnormal white 30-Hz flicker response, a normal blue flash ERG, but a reduced amplitude red flash 30 Hz ERG response to 0.01 red stimuli (Fig. 3D) comparing to normal family member IV-8 (16 year-old-female, Fig. 3F). Moreover, III-11, a 46 year-old female with only eoHM, showed the same red flash ERG response characteristics as IV-10, indicating malfunction specifically in L- and M- cones or their downstream cells (Fig. 3E).

### Data analysis and Sanger sequencing confirmation

We performed the whole-exome sequencing of six affected individuals (II-1, II-3, III-15, IV-1, IV-2 and IV-5) in the multigeneration family. Prioritization steps were applied to reduce the number of variants and identify the candidate pathogenic variants^22^. After the filtering strategy, only c.228T>A (p.Tyr76*) in *ARR3* gene, c.3400A>C (p.Asn1134His) in *PCDH19* gene and c.282C>A (p.Asn94Lys) in *ZNF182* gene were considered as potentially pathogenic variants. Furthermore, the mutation in *ARR3* gene might be responsible for female-limited early onset high myopia. The whole *ARR3* gene was sequenced using Sanger sequencing in all available cases. Additionally, 150 unrelated female patients with a phenotype of high myopia were screened and c.228T>A p.Tyr76* and other pathogenic variants in the *ARR3* gene were absent in the sporadic cases. The p.Tyr76* was not detected in at least 5000 Chinese individuals in the Westlake BioBank for Chinese (WBBC) pilot project^23^, and also absent from the Genome Aggregation Database (gnomAD) database^24^.

To investigate whether male carriers had combined red/green opsin abnormalities, two male carriers, III-1 and IV-9, with color vision defect were examined. Both III-1 and IV-9 were found to possess only one intact copy of both *OPN1LW* and *OPN1MW,* with different variants that were considered from X-chromosome crossing over (Table 2). While III-1 had no harmful variants in *OPN1LW,* a potentially harmful exon 3 haplotype MVVVA in *OPN1MW* was identified, with 75–80% correctly spliced transcript rate^18^. On the other hand, IV-9 presented with a milder color vision defect presentation that mis-recognized only one pseudoisochrmatic chart comparing to other male mutation carriers like III-1. IV-9 shew no harmful variants, but benign exon3 haplotype MVAIS in both red/green opsin genes. These findings suggest that the color vision defects in male family member may be related to both *ARR3* casual mutation and MVVVA haplotype^17,18^.

**Table 2:** Sequence analysis of cloned PCR products encompassing each exons of OPN1LW/OPN1MW genes.

Gene	Position	III-1		IV-9	
		Sequencing results	MUTATION/HAPLOTYPE TYPE	Sequencing results	Mutation/haplotype type
*OPN1MW*	Exon1	Normal		Normal	
	Exon2	Normal		① c.194T>C p.I65T ② c.300G>A p.L100L ③ c.331A>G p.I111V ④ c.347A>C p.Y116S	①Benign ②Synonymous ③Benign ④Benign
	Exon3	① c.465C>G p.V155V ② c.521C>T p.A174V ③ c.532A>G p.I178V	MVVVA (harmful)	c.538G>T p.A180S	MVAIS (Benign)
	Exon4	Normal		Normal	
	Exon5	Normal		c.849A>C p.P283P	Synonymous
	Exon6	Normal		Normal	
*OPN1LW*	Exon1	Normal		Normal	
	Exon2	Normal		Normal	
	Exon3	① c.453A>G p.R151R ② c.457A>C p.M153L ③ c.465C>G p.V155V ④ c.511_513GTG>ATT p.V171I ⑤ c.538G>T p.A180S	LIAIS (benign)	① c.453A>G p.R151R ⑤ c.538G>T p.A180S	MVAIS (Benign)
	Exon4	Normal		Normal	
	Exon5	Normal		Normal	
	Exon6	Normal		Normal	

### Linkage analysis

The results of linkage analysis indicated that the A2-B2-C2-D4-E3-F3-G1-H3-I2, which just labeled around *ARR3* coding region but not including the location of *OPN1LW* and *OPN1MW,* was the disease-causing haplotype which is the same as the genotype of c.228T>A in the family (Fig. 1). All the affected members (II-2, III-1, III-4, III-8, III-15, III-17, IV-1, IV-5 and IV-9) carry the above haplotype while unaffected individuals (III-12, III-13 and IV-8) have the normal one. Linkage analysis showed that the *ARR3 g*ene was co-segregated with high myopia in the family, supporting the pathogenicity of the gene.

## Discussion

**Figure 4:**
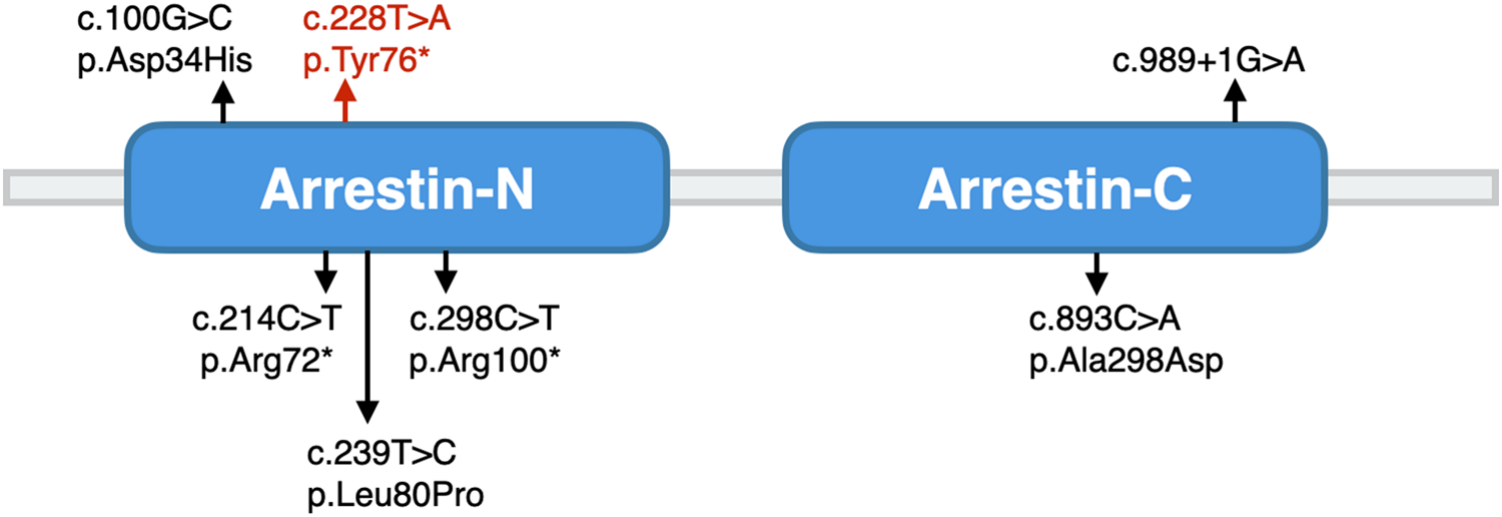
Mutations in ARR3 protein. Mutations in red were identified in the present study, while those in black are from published data.

In this study, we found a family displaying heritable X-link dominant disease including female-limited eoHM and color vision deficiency affecting both sex, carrying nonsense mutation ARR3 c.228T>A (p.Tyr76*). ARR3 causal eoHM is well known for its female-limited transmission in X-linked dominant inheritance. Previously, three Chinese families (c.893C>A (p.Ala298Asp), c.298C>T (p.Arg100*) and c.239T>C (p.Leu80Pro)) and a Hungarian family (c.214C>T, p.Arg72*) showed similar clinical characteristics of female-limited eoHM (Fig. 4)^4–6^. Consistent with these *ARR3*-mutation reports, eoHM was a female-limited manifestation in our pedigree (IV-10, a male mutation carrier, had a history of myopia since Grade 6 in primary school, developing to high myopia in middle school, so was not classified as eoHM).

The presentation of color vision deficiency was not female-limited, thus protan/deutan color vision defect occurred in both female and male mutation carriers, with or without eoHM. The sequencing of red/green opsin genes revealed that this protan/deutan color vision defect may be multivariate that combined *ARR3*-mutation and *OPN1MW* exon 3 haplotype MVVVA in some male carriers like III-1 (Table 2). Haplotype MVVVA has been reported to have 75–80% correctly spliced transcript rate, and is pathogenic in X-linked Cone Dystrophy when present alongside another harmful haplotype LIAVA^18^. Though MVVVA was considered a non-pathogenic variant alone, it is still possible that MVVVA acts synergistically with *ARR3*-mutation in the pathophysiological process of color vision defect^25^. In addition, male *ARR3*-mutation carrier IV-9, with benign opsin haplotype MVAIS, had mild but not typical protan/deutan color vision defect that mis-recognized only one pseudoisochromatic plate, “91”, which was read as “01” (Fig. 2K, Table 2). These finding indicated that *ARR3*-mutation can interfere cone function in color vision of both sexes but differ from that caused by mutated red/green opsins. All family members performed well on the RGB anomaloscope test, except for II-3 who had diabetic macular edema. Further vision tests should be investigated to depict the characters of *ARR3*-caused color vision defects.

Arrestins are modulators of signal transduction which bind active phosphorylated GPCRs, and play a role in receptor-mediated homologous desensitization. Mammals express four arrestin subtypes, all consisting of two domains^8–10^. The *ARR3* gene (also known as cone arrestin, X-arrestin, arrestin-4, or OMIM 301770) is located in Xq13.3 and consists of 17 exons. Its encoded 388-amino-acid protein shows 49% homology with human *SAG* (arrestin protein specially expressed in rod photoreceptors). Figure 4 includes all of the reported *ARR3* causative mutations in the protein structure and all the mutations located in the functional domains^4–6^ and shows that *ARR3* is a relatively conservative gene in functional domain regions.

Shut-off of opsin activity in cone cells occurs more rapidly than rhodopsin recruitment in rods and rhodopsin-like receptors in central neurons, so cones can respond to a 30-Hz flicker in ERG, whereas rods cannot follow a 20-Hz flickering light^19,20^. Absence of both cone and rod arrestin can cause delayed photoreceptor signal shutoff in murine retina, and absence of arr3b has been found to delay zebrafish photoreceptor signal shutoff^26^. In our study, abnormal red flash 30-Hz ERG might reflect reduced speed of opsin shut-off due to dysfunctional *ARR3* in human cones, especially L- and M-cones (Fig. 3). Additionally, relatively normal blue flash 30-Hz ERG may indicate that activated S-opsin is bound by other arrestins in their native condition. Human retina single-nucleus sequencing has shown that cone-arrestin coding by *ARR3* is the most specific molecule marker in cone cells, while rod-arrestin coding by gene *SAG* is not. Rod-arrestin could be detected in some cone cells. It has also been demonstrated that both human and mouse S-cones express both cone- and rod-arrestin^27^ while rod photoreceptors express rod-arrestin only. Thus, when the function of cone-arrestin is changed, its effect differs between cone types, thus M- and L-cone function would change more significantly than that of S-cones.

Due to the different cone transcriptome and function between human and animal models (such as mice and zebrafish), there is still no ideal *ARR3* knock-out model to reveal the pathogenetic mechanism of this female-limited eoHM in various *ARR3* pedigrees^10,12,26^. Széll et al. suggested four potential mechanisms to explain the pathogenic mechanism for eoHM, as follows: (1) a hyperopic defocus based on the differences in sensitized opsins to red/green stimuli caused by abnormal *ARR3* protein function in M- and L-cones; (2) the sensitivity of myopic protective blue light signal may have changed due to cone-arrestin dysfunction; (3) the ocular circadian rhythm changed by intrinsically photosensitive retinal ganglion cells as the result of cone-arrestin dysfunction; (4) change in circadian rhythm due to the expressions of *ARR3* gene in pineal glands^6^. All these hypotheses need to be further tested, and could not explain the sex-separated clinical manifestation. Here, we present our hypothesis based on the relationship between myopia and visional contrast, and the possible pattern of X-chromosome inactivation (XCI) of *ARR3*.

Increasing evidence indicates that malfunction of cones can lead to myopic manifestation. Cases of cone dystrophy, cone-rod dystrophy and color vision defect caused by *OPN1LW/OPN1MW* mutation could have different levels of myopia manifestation^28^. Researchers have investigated the mechanism that splicing defects in opsin genes cause vision disorders, that retinal cone mosaic with adjacent cones differing dramatically in the amount of photopigment signal the presence of constitutive contrast^29,30^. The contrast signal interfered the emmetropization process thus leading to very high degrees of myopia^31^. Moreover, a clinical trial (NCT03623074) of spectacle lenses designed to reduce contrast could reduce myopia progression^32^. This “contrast hypothesis” may also be relevant to *ARR3*-mutated disease. Since arrestins help to terminate the activity of the light activated opsins, aberrated arrestin could not stop the activated signaling and increase the amount of downstream signal. S-cones have both cone- and rod-arrestin, while M/L-cones have cone-arrestin only, so the downstream signal of M/L-cones is increased to a greater extent than that of S-cones. This leads to higher M/L-cone output than in normal conditions, and this aberrated signaling distribution could change visual contrast that associated with myopia development.

The function of M/L- cones in male carriers are all equally impacted due to only one mutant *ARR3* copy, as observed in IV-9, a male carriers who exhibit mild color vision defect that likely was associated with low vision contrast. Such low vision contrast may help to protect male carriers from myopia. For female carriers with two *ARR3* haplotypes, the XCI chooses normal or mutant *ARR3* copy to express while implantation in embryonic period^32^. The mosaic expression of normal or mutated *ARR3* in cones lead to unbalanced cone function that results in higher visual contrast than naturally light input, thereby influencing the severity of myopia and visual acuity loss.

As X-linked dominant disease, female patients usually present variant clinical manifestations because of random XCI. In our pedigree, all female carriers had eoHM except II-2, whereas in other previous reports on *ARR3*-associated eoHM, all female carriers were affected^4–6^. This high penetrance of eoHM in female carriers cannot be explained by randomly XCI alone. Other genetic mechanisms, such as escaping from XCI, may contribute to the disease process and require further explorations.

In summary, our report highlights that clinical phenotype associated with *ARR3* causal mutation can manifest in both females and males with color vision defect and mild cone dysfunction, as well as female-limited eoHM. We also present our hypotheses on the role of aberrated cone output in the mechanism of *ARR3* in pathogenesis.

## Supplementary Information

Supplementary Tables.

## Data Availability

The clinic data analysed during the current study are available from the corresponding authors upon reasonable request. The sequencing data generated during this study are available in the Lei Gu’s repository, https://www.scidb.cn/s/VFjIFv.
