# Supplementary material for: The causal mutation in ARR3 gene for high myopia and progressive color vision defect
Source: Sci Rep. 2023 Jun 2;13:8986. doi: 10.1038/s41598-023-36141-0 (PMC10238396; doi:10.1038/s41598-023-36141-0)
Supplement: Supplementary file 1 — Supplementary Tables. [file 41598_2023_36141_MOESM1_ESM.docx]

**Table S1. The PCR Primer for *ARR3* Gene Coding and Splice Site Region by Sanger Sequencing**

**Name Sequence** **Products length (bp) Tm (**℃**)**

ARR3-exon2_3-F TGTGGGTGAGGTGTCTGTCT 545 60

ARR3-exon2_3-R CCAGACCCCTGAGACCTTGA

ARR3-exon4_5-F TCAAGGTCTCAGGGGTCTGG 536 60

ARR3-exon4_5-R TGGGCTGGAATTCCCAGTTG

ARR3-exon6-F TCTCTGTGTCTTCTTGACCCA 535 60

ARR3-exon6-R CCCTGGCACTACCTATCCCT

ARR3-exon7_8-F GCAAGGTCTCCAGGGAAGAT 538 60

ARR3-exon7_8-R CGATGAGGCTGTGGAGGTTA

ARR3-exon9-F GCCATAGGAAAACAGGCATC 501 60

ARR3-exon9-R CGAATACGCAGATGGAGACC

ARR3-exon10-F GAGACTATGTGCGGCTGGTT 522 60

ARR3-exon10-R AGCCCATGTTAGAGGGAAAGC

ARR3-exon11-F TCACTACCACGGAGAACCCA 506 60

ARR3-exon11-R CACTCTACCCCCTCAACTGC

ARR3-exon12-F GGATGCAACTTACCCCCAGG 499 60

ARR3-exon12-R GAGTCTCTGAACACTGCCCT

ARR3-exon13_14-F GGAATGCATGAGAAGAAACTGG 560 60

ARR3-exon13_14-R CAGACCCTCCAGAATCAATCA

ARR3-exon15-F CAGCCAGGTGAGAGACTGTG 351 60

ARR3-exon15-R GCTATTCCATCCCCAGGCAA

ARR3-exon16-F TTCAGGGAACCGAAGAGCCT 182 58

ARR3-exon16-R TGAAAGCACCCCAAGGAAGC

ARR3-exon17-F GTCCCTTTCAGTGCTCCTGG 532 60

ARR3-exon17-R GGAAGTTGGGGTCAGCGTAT

**Table S2. The PCR Primer for *OPN1LW/OPN1MW* Genes Coding and Splice Site Regions**

| ***PrimerName** | ***Sequence（5'to3'）** | **Products length** | **Tm** |
| --- | --- | --- | --- |
| OPN1MW-E1F | CTGGGATTACAGGTTTCCAG | 533 bp | 60℃ |
| OPN1LW-E1F | GTAGAAGGGAACAGAGAACAC | 620 bp | 60℃ |
| OPN1MW-E1R | GTGTCCTTCTAGATTTGGAG |  |  |
| OPN1MW-E2F | CTGAGGATGGACAAAGCTGG |  |  |
| OPN1MW-E2R | GTAGCAGAGCACGATGATGCT | 4.3 kb | 60℃ |
| OPN1LW-E2R | GTAGCAGAGCATGATGATAGC | 4.4 kb | 60℃ |
| OPN1MW-E4F | CATGCAACATGAATTTCACC |  |  |
| OPN1MW-E4R | CAGAAGCAGAATGCCAGGAC | 2.0 kb | 60℃ |
| OPN1LW-E4R | CAGACGCAGTACGCAAAGAT | 2.0 kb | 60℃ |
| OPN1MW-E5F | GCTGCATCACCCCACTCAGC | 2.0 kb | 60℃ |
| OPN1LW-E5F | GCTGCATCATCCCACTCGCT | 2.0 kb | 60℃ |
| OPN1MW-E5R | GCCTTTCCCGGTCATTTCCC |  |  |
| OPN1MW-E6F | GATGGTCCTGGCATTCTGCT | 2.7KB | 60℃ |
| OPN1LW-E6F | GATGATCTTTGCGTACTGCG | 2.7KB | 60℃ |
| OPN1MW-E6R | TTGCACCCTCTCGAGACCCA |  |  |
| OPN1MW-E2R2 | CAGCCCCACAGCTCCAAACT | 706 bp | 60℃ |
| OPN1MW-E3F | CTCAGTCCGTGGAGCCCTGAATTC | 404 bp | 60℃ |
| OPN1MW-E3R | ACATTGATAGACATTGCACGCTCA |  |  |
| OPN1MW-E4R2 | TGCAGCCACTGGTACAGTCC | 454 bp | 60℃ |
| OPN1MW-E5F2 | GCTCTGGGGTCATCTTAGGC | 608 bp | 60℃ |
| OPN1MW-E6F2 | ATGACATCCCCGCGTGTGAG | 450 bp | 60℃ |

**Table S3. The PCR Steps for *OPN1LW/OPN1MW* Genes Coding and Splice Site Region by Sanger Sequencing**

| **Gene** | **Exon** | **Step 1 for specific separation** | **Step 2 for amplification** |
| --- | --- | --- | --- |
| *OPN1MW* | Exon1 | OPN1MW-E1F/OPN1MW-E1R | The same of Step1 |
|  | Exon2 | OPN1MW-E2F/OPN1MW-E2R | OPN1MW-E2F/OPN1MW-E2R2 |
|  | Exon3 |  | OPN1MW-E3F/OPN1MW-E3R |
|  | Exon4 | OPN1MW-E4F/OPN1MW-E4R | OPN1MW-E4F/OPN1MW-E4R2 |
|  | Exon5 | OPN1MW-E5F/OPN1MW-E5R | OPN1MW-E5F2/OPN1MW-E5R |
|  | Exon6 | OPN1MW-E6F/OPN1MW-E6R | OPN1MW-E6F2/OPN1MW-E6R |
| *OPN1LW* | Exon1 | OPN1LW-E1F/OPN1MW-E1R | The same of Step1 |
|  | Exon2 | OPN1MW-E2F/OPN1LW-E2R | OPN1MW-E2F/OPN1MW-E2R2 |
|  | Exon3 |  | OPN1MW-E3F/OPN1MW-E3R |
|  | Exon4 | OPN1MW-E4F/OPN1LW-E4R | OPN1MW-E4F/OPN1MW-E4R2 |
|  | Exon5 | OPN1LW-E5F/OPN1MW-E5R | OPN1MW-E5F2/OPN1MW-E5R |
|  | Exon6 | OPN1LW-E6F/OPN1MW-E6R | OPN1MW-E6F2/OPN1MW-E6R |
